# ‘It’s nice to just be you’: The influence of the employment experiences of UK military spouses during accompanied postings on well-being

**DOI:** 10.1177/2055102919838909

**Published:** 2019-04-01

**Authors:** Rachael Gribble, Laura Goodwin, Sian Oram, Nicola T Fear

**Affiliations:** 1 Institute of Psychiatry, Psychology & Neuroscience, King’s College London, UK; 2 Department of Psychological Sciences, University of Liverpool, UK

**Keywords:** health and well-being, military spouses, occupational health, qualitative methods, women’s employment

## Abstract

Repeated military relocations (accompanied postings) can have a detrimental effect on employment and well-being among the spouses and partners of military personnel. Semi-structured telephone interviews were conducted with 19 spouses of British Army/Royal Air Force personnel with recent experience of accompanied postings to explore this issue through the lens of self-determination theory; all were married women with at least one child. Participants explained how employment contributed to an independent identity, enabling social connectedness, providing a sense of self-confidence and value but limiting agency over employment decisions. Spouse employment, and therefore, well-being could be improved by the provision of better childcare access or additional support in finding employment and training opportunities.

## Introduction

The career and employment opportunities of the predominately female spouses of US and Canadian Service personnel have been shown to be negatively influenced by the frequent and repeated military relocations (accompanied postings) that are part of life in the Armed Forces (Burrell, 2006; Harrison and Laliberte, 1994; Runge et al., 2014). Accompanied postings are associated with poorer employment outcomes among US military spouses, with spouses less likely to work fulltime and working fewer weeks during the year compared with their civilian counterparts and male military spouses (Cooke and Speirs, 2005; Hosek et al., 2002). Underemployment due to disparities between the educational requirements of the job market and the qualifications held by military spouses can also be an issue, especially in isolated or rural areas with fewer job opportunities (Lim and Schulker, 2010).

How women and families negotiate employment during and between military relocations can influence the well-being of military spouses. Employment among US military spouses has been shown to be important not only for the financial health of military families and personnel retention (Atkins, 2009) but also in providing spouses with a sense of purpose and value and contributing to better well-being and mental health (Faragher et al., 2005; Ickovics and Martin, 1987; Manning and Derouin, 1981; Martin, 1984; Murphy and Athanasou, 1999). US spouses who are able to find work appear to benefit in terms of self-esteem as well as physical and mental health (Klumb and Lampert, 2004; Ross and Mirowsky, 1995; Russo et al., 2000), while unemployment or dissatisfaction with work is associated with poorer mental health and well-being (Faragher et al., 2005; Murphy and Athanasou, 1999; Trewick and Muller, 2014). Despite this body of research, it is unclear whether differences in civilian and military cultures between countries may limit the generalisability of largely US-based research findings to non-US populations.

One theoretical explanation for the impacts of negative employment experiences on military spouse mental health and well-being is self-determination theory (SDT) (Ryan and Deci, 2000). SDT identifies three psychological ‘needs’ that contribute to optimal functioning and well-being: competence/self-efficacy (mastering tasks and gaining new skills), relatedness (connectedness with others) and autonomy (control of behaviours and goals). Social environments that facilitate these needs can positively influence mental health and well-being, while environments that hinder them, such as the hierarchical, authoritarian culture of the military (Knobloch and Wehrman, 2014), may cause greater stress due to a lack of autonomy and motivation (Weinstein and Ryan, 2011), negatively affecting health and well-being. SDT has previously been applied as the theoretical basis for research on health and well-being in occupational research (Gagne and Deci, 2005, Van den Broeck et al., 2016) and more specifically in relation to military personnel and families (Clark et al., 2013; Hodge et al., 2012). However, it has not yet been applied to the study of employment among military spouses.

### Aims of this paper

Within the United Kingdom (UK), there is limited research on the employment experiences of military spouses. Prior studies have focused on overseas postings (Blakely et al., 2014a, 2014b; Jervis, 2011) where employment opportunities are often unavailable to spouses because of restrictions in employment law or language difficulties. It is unclear how the experiences of spouses may differ when families relocate within the UK where such restrictions would not apply or how specifically this affects spouses with children. This study aims to address this gap by exploring the employment experiences of UK military spouses with children during accompanied postings and how these were perceived to have influenced spouse well-being through the lens of SDT.

## Methods

### Participant selection and recruitment

Purposive sampling was used to select spouses/partners of UK military personnel who had recently experienced at least one accompanied posting. Ethical approval for the current study was obtained from the London-Dulwich Research Ethics Committee (NRES reference: 08/H0808/27 Am08-Am09).

Participants were identified from the Children of Military Fathers’ study (Fear et al, 2018), a quantitative study established to explore the impact of paternal post-traumatic stress disorder (PTSD) on the outcomes of children of military personnel. In brief, serving and ex-serving personnel from a large military cohort were invited to participate if they had children aged 3–16 years and according to their scores on the PTSD Checklist – Civilian Version (PCL-C; Weathers et al., 1993). Personnel comprised two groups: the first contained those who met PTSD caseness (score ⩾ 50), were borderline caseness (score = 40–49) or reported at least two of three symptom cluster domains; and the second, those who scored <40 on the PCL-C. Respondents then provided contact details for their spouses/partners, who were invited to participate. Given the aims and design of the Children of Military Fathers’ study, all spouses/partners included in the study had at least one child aged between 3 and 16 years.

Spouses/partners who had consented to follow-up during the Children of Military Fathers’ study were contacted by email or telephone to gauge interest in participation. Spouses/partners of currently serving or former members of the UK Armed Forces who had experienced at least one accompanied posting in the last 5 years or in the 5 years prior to personnel leaving Service were eligible to take part. Attempts were made to recruit a balanced number of participants affiliated with officer and non-officer ranks in order to explore potential differences according to position within the military hierarchy. None of the participants were themselves serving in the UK Armed Forces at the time of interview.

### Data collection and analysis

The semi-structured interview schedule was informed by previous literature, quantitative findings on spouses/partners from the Children of Military Fathers’ study (Gribble, 2017), and key components of well-being as defined in UK public policy – fulfilling their potential, employment, social connections and community (UK Faculty of Public Health, 2010). The interview schedule began with introductory questions to gauge how participants viewed accompanied postings overall, before proceeding to their experiences of employment, family relationships, social support and expectations from the military institution. Within each of these sections, participants were probed on how they perceived their experiences to have influenced their well-being. Spouses/partners of Service personnel who had left Service were asked additional questions relating to their experiences of employment, family relationships and social networks and military support during transition and how they perceived these to have influenced their well-being. Only the findings on employment are reported in this paper. Feedback was provided by representatives of the Army Families Federation (AFF), a military charity that advocates for Army families in the United Kingdom to ensure relevance to military spouses.^1^ Three pilot interviews were conducted with participants to determine question comprehension and the length of interview among spouses/partners. No major changes were made to the interview schedule following the pilot interviews, and they were included in the data analysis.

Interviews were conducted from January to July 2015. As potential participants had families and some were employed and geographically dispersed (including overseas), telephone interviews were selected as the most convenient method for interviewing. A total of 19 participants participated in the study. Interviews were transcribed from audio-recordings. Pseudonyms were used and potentially identifying information removed. Age category, personnel Service branch and personnel serving status are reported alongside illustrative quotes. Quotations are verbatim, with fillers/non-verbal elements removed to improve readability.

Data were analysed using Framework analysis, a structured qualitative methodology that allows responses to be compared across themes and between sub-populations (Lacey and Luff, 2009; Ritchie and Lewis, 2003; Spencer et al., 2003). There are five steps to Framework analysis (Lacey and Luff, 2009; Spencer et al., 2003). The first involves *familiarisation* with the data by reading transcripts, noting common words, ideas or experiences. A *thematic framework* is then constructed either from initial coding or a priori themes from the literature or prior research and applied to the data during the *indexing* phase. The framework is continually developed and refined throughout the following steps. During *charting*, data summaries from each participant are used to outline the experiences and influences on well-being for each participant according to the codes within the thematic framework. The final stage of *mapping* groups the summarised data into similar topic areas (e.g. agency and identity) and sorting into *dimensions* (sub-themes) and *categories* (themes) according to the similarity of their content.

Perceived influences on well-being were identified as descriptions of positive or negative emotional responses to their experiences in accordance with the two continuum model of health and well-being (Westerhof and Keyes, 2010) and discussion of a priori themes of identity, agency/autonomy, relatedness and incorporation from previous spouse employment literature (Enloe, 2000; Finch, 1983; Jervis, 2011); similar aspects were also drawn from SDT. Despite the use of a priori themes, all final themes were developed using iterative and inductive techniques based on the data collected. Themes and sub-themes were checked by a second coder (G.K.T.) and feedback from AFF representatives was used as a form of participant validation. Comparisons between influences of well-being according to personnel rank (officer/non-officer) and Service (Army/Royal Air Force (RAF)) were planned prior to data collection and are reported where relevant.

## Results

### Description of the sample

A total of 19 interviews were completed with women married to current or former members of the British Army or RAF of Sergeant rank or above (Table 1). As a result of the original study used for recruitment, all had at least one child ranging in age from 2 to 27 years, with most aged between 5 and 12 years. All participants had experienced at least one accompanied posting in the last 5 years or in the 5 years prior to personnel leaving Service.

**Table 1.** Qualitative study participants.

Rank	Age (years)	Pseudonym	Personnel Service branch	No. of years married	Total no. of postings	Occupational area
NCO	30s	Courtney	RAF	5–9	<5	Teacher
		Mary	Army	10–14	<5	Health services
		Allison	Army	10–14	5–9	Educational support
		Dee	Army	15–19	5–9	Educational support
	40s	Molly	Army	5–9	<5	Educational support
		Gina	RAF	10–14	<5	Teacher
		Janet	Army	15–19	<5	Health services
		Linda	Army	20+	5–9	Stay-at-home parent
Officer	30s	Jennifer	Army	10–14	5–9	Educational support
		Louise	RAF	10–14	5–9	Self-employed
	40s	Toni	RAF	10–14	<5	Teacher
		Anna	Army	10–14	5–9	Health services
		Joan	Army	15–19	5–9	Health services
		Kathleen	Army	20+	5–9	Stay-at-home parent
		Kim	Army	20+	5–9	Charity
		Suzy	Army	20+	10+	Financial services
	50s	Carrie	Army	20+	5–9	Self-employed
		Kristen	Army	20+	10+	Educational support
		Melissa	RAF	20+	10+	Corrections services

NCO: non-commissioned officer; RAF: Royal Air Force.

### Influence of spouse employment experiences during accompanied postings on well-being

**Figure 1.**
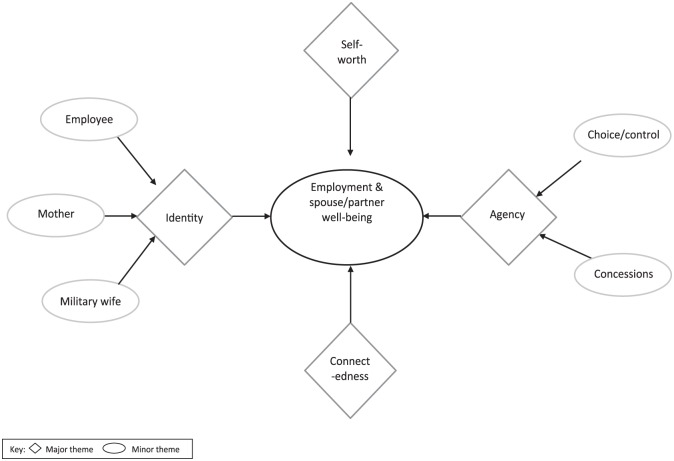
Thematic diagram of the influence of spouse/partner employment experiences during accompanied postings on well-being.

Four major themes were identified (Figure 1): identity, agency, self-worth and connectedness.

#### Identity

Three types of social role were identified within this theme: *employee, military wife* and *mother*. Employment – and the identity of ‘employee’ obtained through work – was described by a number of participants as a positive influence on their well-being, enabling spouses to reclaim a sense of independence and self beyond the military community and contributing to their self-image. Some spouses described tensions attempting to balance these three competing identities that could influence well-being. Spouses reported feelings of guilt when trying to manage work and family life alongside employment, while others reported a perceived loss of status when unable to work.

##### Employee

Some spouses explicitly described employment as an integral component of their self-construction and identity. This was not reported to be in relation to any particular profession or occupation but rather to the identity spouses obtained as a result of work. Work provided a sense of stability or security for the identity of some spouses – ‘*your job becomes part of your identity*’ (Kim, 40s, officer, Army) – and therefore became a strong motivation for seeking employment:

… [work] is part of my identity … it is part of who I am … the move that I found most difficult was when I was on maternity leave … after that maternity leave I think I really wanted to just get back to a little bit of who I am and what I know. So going back to work was really important. (Anna, 40s, officer, Army)

For other spouses, employment was less about providing an identity or status and instead related to a sense of purpose and structure to their lives. Such participants were largely content to take any form of employment that allowed them to feel they were productive outside the family home:

… I’ve been working six months since moving up here and I’ve taken a down step … from a well-being point of view, it’s good because I’d rather be out doing something of a lower pay, a lower grade, meeting people in the community, rather than being at home. (Molly, 40s, NCO, Army)

In either situation, the inability to find work following accompanied postings was described as influencing how some spouses perceived their value and status within society. Participants who found employment in roles that they felt under-utilised their skills or experience felt they were not sufficiently financially or professionally recognised or rewarded. Others described how they perceived there to be a reduction in their ability to support and provide for their families which could contribute to negative moods and emotions:

I felt like I’ve been getting employed on the cheap. And I recognise now that whenever I move that potentially could always happen … that’s a big deal for me I feel! (Courtney, 30s, NCO, RAF)

… [not working] … affects my moods … I get quite down about it and because I like to be able to do something) to contribute even if it … buy the week’s shopping. … it’s something and it does get me down. (Dee, 30s, NCO, Army)

##### Military wife

Many spouses described how their relationship with their husband led them to be ascribed the identity of a ‘military wife’ or dependent within the community. The imposition of this identity could become a driver to seek out employment among spouses who were resistant to being linked with their husband in this way. By establishing connections and relationships with civilians, spouses were able to reassert their independence and resist the identities imposed on them through their relationship with their husband within their working environment:

… it’s nice to just be you, just be Allison and not ‘Oh your husband’s [rank] [surname]’ … you’re your own person and you’re not sort of classed with your husband as such … that’s nice. (Allison, 30s, NCO, Army)

… if I didn’t work it would drive me mad! … Because I think I have lost a bit of my identity being married to someone in the military. (Gina, 40s, NCO, RAF)

Spouses who disliked this ascribed identity but were unable to find employment, for example, during overseas or short postings, described how being viewed as a ‘military wife’ challenged their perceived independence and status within the community that they had previously obtained through their own employment:

… [I felt] bored! Frustrated … kinda a bit useless really. I just felt like that spouses on someone’s arm … I like to go and make my own money. I don’t like to rely on people and I had to rely on my husband for kind of everything out there. (Mary, 30s, NCO, Army, transitioned)

… you get to [Europe] and then you become a dependent. And basically you lose any status you’ve ever had and you have to go through a [job search] process which is … quite demeaning … I was quite high [up] where I was and I’m suddenly having to do typing tests to even get into their pool of employees. (Molly, 40s, NCO, Army)

Not all participants resisted taking on the identity of a military wife, with some able to use this identity to find employment and volunteering opportunities within the military community. In doing so, spouses were able to mitigate the negative effects of accompanied postings on their own employment and well-being and gain a sense of connection, purpose and value through employment within the community:

… the job I currently do now I did … I’ve done in [multiple] locations. I actually work for [welfare service] … that is probably what really helped me because I was able to use, and I still do today … I use my experience in the Army to help people every day. (Kim, 40s, officer, Army)

##### Mother

All participants in this study had children. Many working spouses reported difficulties in balancing their family responsibilities with employment due to the cost and availability of formal childcare and – because of geographic distance from family members during accompanied postings – a lack of informal support. For some spouses, this could lead to internalised conflict about whether or not they believed they were adequately fulfilling their responsibilities as ‘good’ mothers. Some described feeling divided between the competing demands of parenting and work, and guilt about a perceived failure to adequately balance the two. This conflict was largely expressed by spouses of officers but was related to difficulties balancing career aspirations with family life rather than to their husband’s position in the military hierarchy:

… working now, I don’t feel like I do my mum bit as well as I did. And I feel guilty about that … I’m conscious that I’m … not 100% there for them … when they’re home I want to be at home, but I’ve got to be working. (Suzy, 40s, officer, Army)

Not every spouse was interested in prioritising their career and military life could provide spouses with an opportunity to establish more traditional family structures. Some spouses saw themselves as a mother first and were content to form their identity around this role rather than that of an ‘employee’:

… [when] we’ve been in the military has been the time when I’ve had children … from my point of view, it’s been quite a release … because of the moving around I’ve not even thought about the whole ‘shall I go back to work’ thing … actually I’m glad I’ve not had to think about it! … I wanted to be at home for [the children] and … [not] worrying about when I was going back to work. (Kathleen, 40s, officer, Army)

… [children] were always going to interfere with my career … we made a decision when I had our children that I would give up work until they went to secondary school … I’d have probably made that decision anyway … (Suzy, 40s, officer, Army)

#### Agency

Military life was reported to constrain spouses’ agency with regard to planning and making decisions about their employment, with implications for job satisfaction, anxiety, stress and resentment towards the military. This theme comprised two sub-themes: *choice/control* and *concessions*.

##### Choice/control

A prominent view among spouses was that accompanied postings limited their ability to have choice and control over their career or employment. As a consequence, spouses were unable to plan ‘career paths’; spouses who wanted to work described this as negatively affecting job satisfaction and generating resentment. In some cases, this had serious implications for the emotional well-being and mental health of spouses who were not able to overcome the obstacles they encountered:

I’ve grabbed every opportunity I’ve been able to, but … there’s not really been my conscious choices … [I feel] a little bit resentful because I think we’ve got into a mode now where … I’m working for the money and just keeping in employment, but not really enjoying it too much! (Gina, 40s, NCO, RAF)

… I desperately wanted to work and I did find a job eventually. But I found myself in a real sort of trench of depression because I just couldn’t see where my life was going I suppose. (Carrie, 50s, officer, Army, transitioned)

Anticipation of a loss of control preceded accompanied postings for some spouses; uncertainty regarding work could lead to worry and anxiety during this period, especially in relation to the potential financial implications for the family. These feelings were particularly acute among spouses for whom previous accompanied postings had had negative impacts on their employment:

… It is quite an unsettling feeling … you’ve got all the worries of looking and starting all over again really … You try not to get you know sort of stressed about it, but it’s just something that happens … you get used to that income a month and then it’s like … ‘When can I start looking?’ and ‘Am I going to get anything?’ … I get quite anxious. (Allison, 30s, NCO, Army)

##### Concessions

As well as limitations on active employment choices, some spouses described the sacrifices they were required to make regarding employment or education because of the restrictions encountered through their contact with the military. When speaking about these concessions, some women expressed feelings of resentment, frustration and unrealised potential. As with tensions between the roles of employee and mother, discussions of unrealised potential in this sample were dominated by the spouses of officer personnel; this appeared to be related to motivations for work or education, with these participants the most eager to maintain or build a career:

… there is no career. It is about getting bits and bobs of jobs that I can get … on a good day I kind of accept that for being in the military and a military wife and on a bad day it, it’s more difficult to swallow … it does feel like a bit of a kick in the teeth having [a job] that effectively I could have done ten years ago … it just feels that you’ve sacrificed … an awful lot … (Joan, 40s, officer, Army)

… Very frustrated at times. Very frustrated, feeling like the inner flexibility, if you know what I mean, with the Army and where he could be posted meant that I couldn’t achieve something for me. And at that time it meant a lot to me. (Kim, 40s, officer, Army)

The concessions spouses made to their employment also had consequences for their financial situations. Some spouses described how limitations to their employment compromised their financial independence and necessitated their reliance on their husbands for money during times of unemployment. For some, this was a minor issue that was resolved once they found work but others reported how prolonged unemployment and the resulting loss of access to money they had earned themselves was important not just for a sense of achievement but for maintaining a sense of independence:

I like to go and make my own money. I don’t like to rely on people … [I worked] so that I could … I’d just have my bit of money. It was mine that I’d earnt and just to have something for myself really. I didn’t really have anything for myself. (Mary, 30s, NCO, Army, transitioned)

#### Self-confidence and connectedness

Two other themes were also identified: self-confidence and connectedness. The experiences spouses had of employment during accompanied postings had different influences on how spouses viewed themselves in terms of self-worth. For some spouses, finding work after taking time out to raise children could be a time of anxiety and uncertainty, affecting confidence in their skills and experience. However, for others, overcoming barriers and challenges to employment that they encountered were seen as contributing to personal growth and confidence. As with concessions, this theme was largely discussed by the spouses of officers with a strong desire to work:

… when I went back to work after eleven years out of the workplace it was quite daunting in terms of my confidence. Even though I had the academic ability on paper it didn’t feel like it. And even though I know I had a brain, it didn’t feel like it inside. (Suzy, 40s, officer, Army)

… [moving] … has actually made me in some respects a stronger person, a more independent person … it can also help you be more focussed … on what you do … sometimes you have to look outside the barriers … and work around it. So that can make you stronger. (Kim, 40s, officer, Army)

Another important function of employment was in providing spouses with a means of developing social connections. Some spouses reported how work benefitted them by providing social avenues in which they could meet people following an accompanied posting to a new area where they may not know anyone. Such relationships and interactions were reported to have a positive influence on spouse well-being, especially where it allowed them to meet with other people outside the family home:

… for me in terms of mental health, working does … has been of great benefit in terms of it allows you to get to know people … after a move … it gives you the shared experience … (Anna, 40s, officer, Army)

… it’s quite nice to be able to talk to other adults as well. I think I’d go insane if I just stayed at home all the time! (Jennifer, 30s, officer, Army)

## Discussion

This study explored how the employment experiences of spouses of UK Armed Forces personnel during accompanied postings were perceived to influence their well-being. The identified themes reflect how the three psychological ‘needs’ of military spouses outlined in SDT – competence/self-efficacy, relatedness (connectedness) and autonomy (agency) – are hindered or facilitated by the employment experiences of spouses during accompanied postings. The themes also align with elements of eudemonic well-being: self-acceptance, positive relations with others, autonomy, environmental mastery, purpose in life and personal growth (Ryff, 1989; Ryff and Singer, 1996). While the findings highlight the challenges many military spouses face in finding and maintaining employment across relocations and the influence of these difficulties on their sense of identity and agency, there could also be benefits. Overcoming these challenges was important for some spouses to maintain a sense of identity or choice, whereas other spouses who were able to overcome perceived barriers and challenges to employment described positive influences on their well-being due to improved self-confidence and increased access to social connections

Spouses in professional careers (also typically the spouses of officers) described how they were intent on working during accompanied postings because of the meaning it provided them with in (re-)establishing an identity separate to that of military wife or mother. Yet, they experienced difficulties planning or progressing their careers because of the frequency and duration of accompanied postings. Spouses who were not in professional careers reported how they were motivated to work by the sense of purpose, self-confidence and affirmation of value they obtained, which corresponds to the commonly cited reasons for working among US spouses (Castaneda and Harrell, 2008; Maury and Stone, 2014; Peterson, 2002). For both groups of spouses, the centrality of employment to their identity construction (Bothma et al., 2015) was difficult for them to balance against the compromises and sacrifices they felt they required as ‘good’ military wives (Enloe, 2000). As this study suggests, spouses for whom employment is important for either status or identity but who are unable to obtain appropriate employment may be at higher risk of poor outcomes such as low self-esteem and psychological distress (Sowislo and Ulrich, 2013).

Spouses with professional careers described how unemployment or employment that they felt under-utilised their skills and experience contributed to a perceived loss of social status both within their communities and their relationship. This sense of a loss of status due to a suspended or halted career has also described by spouses on overseas postings (Blakely et al., 2014b; Jervis, 2011) and spouses within expatriate communities (Cieri et al., 1991). The sense of financial independence that spouses, both in professional and non-professional occupations, gained from employment was described as another loss related to accompanied postings. This loss was not related to money per se but to the sense of achievement and pride spouses gained from earning their own money and contributing to their family. Adjusting to the loss of their income, which represented independence to some spouses, was difficult for these women (Enloe, 2016; Harrison and Laliberte, 1994; Horn, 2010).

Although many participants described frustration at the lack of agency and choice they exercised over their employment and careers, there was variation in the extent to which spouses prioritised employment over being a fulltime parent. Spouses’ well-being has been shown to improve with greater satisfaction with their daily role, regardless of whether that is as a stay-at-home parent or as an employee (Rosen et al., 1990). Caring for children is a major reason for economic inactivity among US spouses (Blue Star Families, 2014; Harrell et al., 2004; Maury and Stone, 2014) and has been shown to be associated with lower dissatisfaction with employment in limited or competitive labour markets (Cooney et al., 2009). Similar findings are seen in this study among participants who expressed little or no motivation to seek employment, when their current role as a stay-at-home mother provided them with a strong, alternative identity to that of ‘employee’.

Despite the employment difficulties spouses encountered, overcoming the challenges of finding employment was described by some spouses as having boosted their self-esteem and provided an opportunity for establishing social connections following an accompanied posting. Studies have suggested that good quality and supportive relationships can be difficult for military spouses to build and maintain because of frequent moves (Finch, 1983; Jervis, 2011; Orthner and Rose, 2009; Padden and Posey, 2013) and that spouses who are unable to build connections within the military community may be at greater risk of social isolation, stress, psychological distress and mental health problems (Cohen and Wills, 1985; Dalgard et al., 1995; Greenblatt et al., 1982; Knickmeyer et al., 2002; Maulik et al., 2010; Olstad et al., 2001; Padden and Posey, 2013; Paykel, 1994). Findings from this study suggest that spouses who are able to work may be better able to mitigate the disruptive social impact of accompanied postings through the development of new networks, potentially improving mental health and well-being.

## Strengths and limitations

This is the first UK study to examine the employment experiences of military spouses during both overseas and UK accompanied postings and how these can influence well-being, deepening current knowledge in this area. Other qualitative studies of employment among UK military spouses have been conducted (Blakely et al., 2014a, 2014b; Jervis, 2011) but have only focused on the influences of overseas postings on spouse well-being. Differences and similarities were explored according to personnel rank and Service branch to examine how military hierarchy and structure may influence spouse well-being. While there are criticisms of the use of telephone interviews, this method can provide social and physical distance from the interviewer, which can result in participants being more open in relation to potentially sensitive issues, such as relationships and financial issues as discussed in the interviews (Greenfield et al., 2000; Mealer and Jones, 2014).

Although this study provides insight into the experiences of spouses of UK military personnel, the findings may not be transferable to spouses who were not represented in the participant sample. As participants were recruited from the Children of Military Fathers’ study (Fear et al., 2018), all participating spouses had children, who varied in age. Therefore, these findings may be less applicable to women who do not have families or whose children have left home and may reflect, in part, adjustment to being a mother within the military community. As all participants had been married for at least 5 years, with some married for more than 20 years, the sample is likely to be biased towards couples that have adapted to military life and the demands of frequent relocation. Future research should explore these findings among other groups of spouses and partners, such as newly married military couples, spouses/partners without children, spouses/partners of Royal Navy/Marine personnel, spouses/partners of personnel of Sergeant rank and below and unmarried partners.

## Implications

Encouraging and supporting spouses to not only obtain work but also obtain fulfilling employment could result in improved well-being among this population, with higher levels of well-being found among people who reported meeting needs of autonomy (control of behaviours and goals), competence (mastering tasks and gaining new skills) and relatedness (connections with others) during past events (Phillipe et al., 2011, 2012; Waters, 2014). Such perceptions are important to consider, particularly in light of the spousal employment component of the recent UK Ministry of Defence’s Armed Forces Families Strategy (Ministry of Defence, 2016) and associated programmes. However, while this study focused on the spouses of military personnel, the findings are also applicable to other occupations that involve a high level of mobility, such as the families of diplomats or financial employees moving between international offices, especially when the majority of UK families are now dual-income (Office for National Statistics, 2017).

Spouses who were able to pursue their careers or find fulfilling employment described how employment improved their well-being by contributing to an identity independent of their roles as military wives and provided a sense of purpose and value. The use of employment to establish social connections in a new area could also assist spouses in alleviating the disruptive impact of accompanied postings on social networks, potentially improving mental health and well-being and reducing psychological distress.

Recent attempts have been made by the Ministry of Defence to improve employment outcomes among military spouses/partners to address these challenges and improve spouse and family well-being. Spouse/partner employment has been included as a major component of the Armed Forces Families’ Strategy (Ministry of Defence, 2016), and a spousal employment support trial is currently underway in Cyprus to help spouses find fulfilling employment (Career Transition Partnership (CTP), 2015). However, more targeted support could be provided. Additional targeted support could be provided through existing employment and training for spouses who would particularly benefit from work, such as those who view employment as important to their identity and those less likely to be working such as spouses/partners of Army, lower ranked personnel and those living on/near military bases (Gribble, 2017). Given the participant sample of spouses/partners with children, childcare was a major issue in relation to employment. Military welfare should also consider ways of improving provision of and access to childcare for both Service personnel and spouses/partners, with more flexible opening hours to counter the lack of informal care from family members as a result of accompanied postings. Providing increased flexibility in their working arrangements as well as allowing them to pursue further education to update their skills and qualifications if required, relieving some of the anxiety, stress and guilt spouses may experience when attempting to balance the demands of work and family life.

## Conclusion

Employment performed several roles for the spouses of UK military personnel during accompanied postings that aligned with the psychological needs described by SDT: contributing to an identity independent of their roles as military wives and mothers; enabling social connections; and providing a sense of purpose and value. Spouses with a strong desire to work or those associating employment with social status may be at a higher risk of low self-esteem and psychological distress. While attempts have been made to address this issue, military welfare policy should consider additional ways of supporting spouse employment and improving well-being outcomes, such as by providing practical support for childcare provision or additional guidance for spouses in finding employment and training opportunities.

## Notes

1. See www.aff.org.uk for further information.
